# Predicting Deterioration from Wearable Sensor Data in People with Mild COVID-19

**DOI:** 10.3390/s23239597

**Published:** 2023-12-04

**Authors:** Jin-Yeong Kang, Ye Seul Bae, Eui Kyu Chie, Seung-Bo Lee

**Affiliations:** 1 Department of Medical Informatics, Keimyung University, Daegu 42601, Republic of Korea; jykjin@yonsei.ac.kr; 2 Department of Statistics and Data Science, Yonsei University, Seoul 03722, Republic of Korea; 3 Department of Family Medicine, Kangbuk Samsung Hospital, Sungkyunkwan University School of Medicine, Seoul 03181, Republic of Korea; ys13.bae@samsung.com; 4 Department of Future Healthcare Planning, Kangbuk Samsung Hospital, Sungkyunkwan University School of Medicine, Seoul 03181, Republic of Korea; 5 Department of Radiation Oncology, Seoul National University College of Medicine, Seoul 03080, Republic of Korea; ekchie93@snu.ac.kr

**Keywords:** monitoring, wearable sensors, machine learning, mild COVID-19

## Abstract

Coronavirus has caused many casualties and is still spreading. Some people experience rapid deterioration that is mild at first. The aim of this study is to develop a deterioration prediction model for mild COVID-19 patients during the isolation period. We collected vital signs from wearable devices and clinical questionnaires. The derivation cohort consisted of people diagnosed with COVID-19 between September and December 2021, and the external validation cohort collected between March and June 2022. To develop the model, a total of 50 participants wore the device for an average of 77 h. To evaluate the model, a total of 181 infected participants wore the device for an average of 65 h. We designed machine learning-based models that predict deterioration in patients with mild COVID-19. The prediction model, 10 min in advance, showed an area under the receiver characteristic curve (AUC) of 0.99, and the prediction model, 8 h in advance, showed an AUC of 0.84. We found that certain variables that are important to model vary depending on the point in time to predict. Efficient deterioration monitoring in many patients is possible by utilizing data collected from wearable sensors and symptom self-reports.

## 1. Introduction

The COVID-19 pandemic has caused over 6.9 million deaths [1]. In addition to the causative virus severe acute respiratory system, SARS-CoV-2 can cause complications in other organ systems (e.g., cardiovascular, nervous, renal), which can also contribute to death from this disease [2]. Since mortality in the severe group is 49% and they are at high risk of complications such as severe pneumonia, acute respiratory distress syndrome, septic shock, and organ failure [3,4,5], severe patients with COVID-19 often receive special care in isolation facilities within hospitals. Therefore, many prior studies focus on severe groups to predict mortality or prescreening using initial clinical symptoms [6,7,8].

From a management perspective, patients with low severity, that is, asymptomatic or mild COVID-19 patients, do not require any special management other than quarantine or management, such as sufficient rest [9]. However, some asymptomatic or mild COVID-19 patients may experience a rapid deterioration within a few hours, necessitating transfer to an intensive care unit for critical treatment [5]. Therefore, early identification of COVID-19 patients at risk of severe illness is critical to identifying which patients will receive priority treatment, and early prediction can allocate medical resources cost-effectively and potentially reduce fatality rates [10,11,12]. There are some indicators of deterioration, such as the early warning score (EWS), but they are not suitable for large-scale observations. A monitoring solution for all patients with mild conditions is needed with a limited budget and management staff [13]. In a way that uses fewer management staff, several studies have attempted to predict the deterioration of COVID-19 patients. A previous study defined deterioration based on a commonly used risk score for early recognition of patients with severe infection and developed models to predict deterioration using machine learning methods [14,15,16,17]. A limitation of this study is that it was conducted only on hospitalized patients. For those under self-quarantine, visiting a high-level hospital means that they have already experienced a clinical deterioration. There are also studies conducted on non-severe people, but these studies also used data that were difficult to measure easily, such as lab tests and computed tomography (CT) [18,19]. These studies are inadequate to respond to the trend of the spread of infectious diseases, with the number of mildly ill patients increasing. Therefore, a more popular deterioration prediction method that can be used by many people is needed.

Currently, with the growth in sensor technology and the decreasing cost of wearable sensors, monitoring patients using biometric data such as body temperature, respiratory rate, and heart rate measured from wearable devices has been commercialized [20]. Wearable devices have the advantage of being able to safely and continuously monitor low-risk patients at a relatively low cost because they can be continuously attached to the patient’s body and measure vital signs. Accordingly, studies have been conducted to detect infectious diseases, influenza [21], COVID-19 [20,22], and so on [23] using wearable devices. However, most of those studies require a long measurement time, and performance measurements were performed in experimental settings, not in actual clinical environments. In addition, some patients show characteristics of repeated worsening and improvement [24,25], and in the case of mild patients with COVID-19, indirect measures such as non-face-to-face treatment can be taken rather than active measures such as immediate transfer as the possibility of deterioration is high. Above all, previous studies focused on detecting the presence of the disease, and the situation of predicting clinical deterioration in real time was not considered, so the potential of a system using a wearable device that can monitor continuously was not fully utilized.

In this study, we propose a machine learning based modeling approach for the prediction of clinical deterioration using data that were easy to measure. Based on previous research related to predicting deterioration of COVID-19 patients [14,17,26], we proposed a fast and interpretable deterioration detection model using four algorithms: random forest (RF) [27], eXtreme Gradient Boosting (XGB) [28], light gradient boosting machine (LGBM) [29], and CatBoost algorithms [30]. This study was conducted to prepare for a large-scale infection situation by analyzing the general public with mild COVID-19 and conducting an evaluation that considered the actual situation by measuring data in real-time and deriving predictions about clinical deterioration. The proposed model predicts the deterioration of mild COVID-19 patients for two scenarios: a 10 min advance prediction model for responding to deterioration within medical facilities and an 8 h advance prediction model for responding outside of medical facilities.

## 2. Materials and Methods

### 2.1. Study Design and Population

This retrospective study was conducted at Seoul National University Hospital (SNUH). This study obtained approval from the Institutional Review Board of SNUH (IRB number: H-2105-158-1221). The study cohort consisted of patients who were aged 18 years or older and diagnosed with COVID-19. The derivation cohort consisted of people diagnosed with COVID-19 between September and December 2021, and the external validation cohort consisted of people diagnosed with COVID-19 between March and June 2022. COVID-19 was diagnosed using real-time reverse transcription polymerase chain reaction (RT-PCR) testing at local health centers. During the middle phase of the pandemic, from the second half of 2021, all mild clinical cases were quarantined in their homes, and severe patients were transferred to hospitals in accordance with Korea’s COVID-19 patient management guidelines. All patients participating in this study had mild or asymptomatic symptoms and were quarantined at home according to these guidelines. Even patients in self-quarantine often had pain, such as high fever or severe sore throat, and data in this study were derived from a study conducted to manage and monitor these patients effectively.

### 2.2. Clinical Data Acquisition

Patients who participated in this study were instructed to wear wearable devices all day and answer clinical questionnaires twice daily while in quarantine. These data were recorded with two types of wearable devices: Garmin Venu sqr (Garmin Inc., Olathe, KS, USA) in the form of a wristband and mobiCARE+Temp MT100D (Seers Technology, Seongnam-si, Republic of Korea) in the form of a patch. Only body temperature was measured on the wrist with a patch-type device, and heart rate per minute, respiratory rate per minute, and saturation pulse oxygen (SpO_2_) were measured with a wristband-type device. Body temperature, respiratory rate, and SpO_2_ were usually measured at 1 min intervals and heart rate was measured at 15 s intervals. Features extracted from wearable devices used statistical values (mean, median, maximum, minimum, and standard deviation) within the observation window.

When collecting the derivation cohort, all patients had to receive non-face-to-face treatment from medical staff at least twice daily, and medical staff recorded the patients’ self-measured blood pressure, respiratory rate, oxygen saturation, and symptoms complained of by the patient. However, when collecting the cohort used for external validation, patients self-reported clinical questionnaires twice daily. The questionnaire items were mainly related to the symptoms currently being experienced and determined through research and research conducted early in the pandemic [31,32]. The collected symptoms are shown in Table 1. The clinical questionnaire concatenated that response times were at or before the ranges.

### 2.3. Definition of Deterioration

Our main goal is to predict deterioration in advance, so we set two prediction times: 10 min in advance and 8 h in advance. Deterioration was defined as a body temperature above 37.5 °C in this study [24]. Since the temperature measured at the wrist is slightly lower than other parts and because these models predict the possibility of deterioration rather than high fever, the threshold of body temperature is lower than in other studies [33].

### 2.4. Extraction of Outcomes and Features

The prediction model aggregated data over a certain time interval and then calculated the risk of deterioration after the forecast periods. The features used in the deterioration prediction model are measured using wearable devices and patients’ symptoms. Even though the features were measured using the same device, the time intervals were different, so the time range was based on values measured by the patch. 

**Figure 1:**
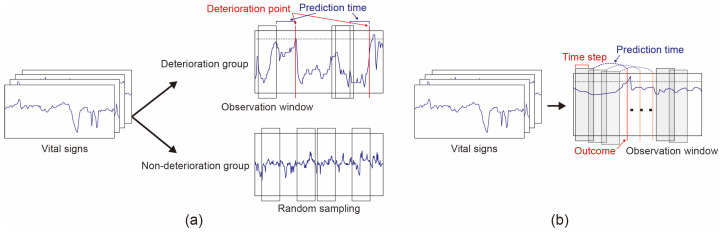
Illustration of the feature extraction step from vital signs. (**a**) The process of extracting variables used for training models. Separating patients who experienced deterioration from those who did not and an observation window moved forward the prediction time; (**b**) The process of extracting variables used for model evaluation. An observation window moved one-time step through each patient’s signal data.

A detailed process of feature extraction is shown in Figure 1. For the deterioration group in which deterioration was observed more than once during the isolation period, features were extracted based on the time of deterioration. A model that predicts events after T-hours using an observation window of N-hours used features extracted from vital sign records between (T + N) hours and T hours before the event. For example, the deterioration prediction model 8 h in advance used features extracted from the measurements between 9 and 8 h before deterioration. For the non-deterioration group, the same method was applied to 500 randomly selected times. In the training set of the deterioration prediction model 10 min in advance, there are 68,881 observations with a deterioration class and 90,757 observations with a non-deterioration class. In the deterioration prediction model 8 h in advance, there are 56,144 observations with the deterioration class and 69,188 observations with the non-deterioration class. It was not possible for us to adjust the number of observation deterioration data, but we could adjust the non-deterioration observation, so we chose 500 points to ensure that the two classes were balanced. Considering class imbalance and usability in clinical settings, the observation time was set to 1 h. 

The external validation dataset was composed in a different way from these training data to evaluate the model’s performance in a clinical environment. These data were aggregated using the same observation window based on the measurement by patch. An N-hour observation window moved forward every 10 min, and a prelabel was assigned according to the maximum value during the observation period. The final label is given by shifting the prelabel by the prediction time. In the training set of the deterioration prediction model 10 min in advance, there are 4925 observations with a deterioration class and 65,168 observations with a non-deterioration class. In the deterioration prediction model 8 h in advance, there are 3927 observations with the deterioration class and 62,062 observations with the non-deterioration class. Since the time when deterioration is detected is significantly less, in reality, the dataset was imbalanced, with most samples belonging to the non-deterioration label. 

### 2.5. Development of the Model 

The goal is to build a binary classification model that predicts whether a COVID-19 patient will have a deterioration after prediction. Five-fold cross-validation based on patient number was used for model development. We divided these derivation data into five folds, repeatedly trained on four folds, and tested with the remaining fold. Each fold was arranged so that the number of patients who experienced deterioration was similar to ensure balanced data organization. We ensured that data from one patient was not placed in a different fold. Each fold consists of 5 or 6 people who experienced deterioration and 4 or 5 people who did not experience deterioration. The results were evaluated based on the area under the receiver operating characteristic curve (AUC), and predicted values were evaluated after integrating the folds. After developing models trained on derivation data, we evaluated them by applying them to an external validation dataset. We compared the performance of four machine learning algorithms, RF, XGB, LGBM, and CatBoost, to determine which algorithm fits our data. Then, we selected subsets of features using a recursive feature elimination algorithm [34]. Classification models were trained using selected subsets and evaluated using various metrics to find the optimal combination. The local interpretable model-agnostic explanation (LIME) method was used to identify features that affect fever and different patterns depending on the time period to predict across the entire feature space of the final model [35]. A final model using minimal features identified meaningful clinical differences between patients.

### 2.6. Statistical Analysis

Data collected on the first day of admission were analyzed using Pearson’s chi-square test to compare the differences between patients who experienced deterioration during the measurement period and the other group. Continuous variables were non-regularly distributed and were compared using the Mann–Whitney U test. COVID-19 symptoms were analyzed and compared between cohorts and between groups that experienced deterioration and those that did not use the chi-square test. The AUCs, accuracy, sensitivity, specificity, positive predictive value (PPV), and negative predictive value (NPV) were compared to evaluate the discriminatory power of the models. Except for the AUCs, the evaluated values used the cut-off value of Youden’s index [36]. When comparing the AUCs between the models, these data were resampled using the bootstrap method, and the average and variance of the AUCs were calculated. The DeLong test [37] was performed to compare the predictive abilities of models that used different feature combinations. All tests were two-sided, and *p* < 0.05 was considered statistically significant. All statistical analyses were conducted using Python v3.8.8 and SciPy v1.5.2.

## 3. Results

### 3.1. Demographic and Clinical Characteristics

The derivation cohort consisted of people diagnosed with COVID-19 between September and December 2021. Data were collected for a maximum of 9 days and a minimum of 2 days from a total of 50 patients. The age of the 50 patients ranged from 20 to 66 years, with a mean age of 39 years (SD 13.2). Patients wore the smart watch-type wearable device for an average of 79.5 h (SD 45.3 h) and the patch-type device for an average of 73.9 h (SD 42.8 h) during isolation. The total time the deterioration was detected was 51.2 h, and the average was 1.8 h per patient. Twenty-eight patients (56%) experienced deterioration during isolation. All patients were primarily screened; none had severe dyspnoea uncontrolled by medication. The general clinical characteristics of the derivation cohorts collected at initial diagnosis, stratified by with and without deterioration, are summarized in Table 1. The group that experienced deterioration during isolation had a higher pulse rate and temperature than the group that did not and responded that they had pain when filling out the clinical questionnaire.

### 3.2. Comparison of Predictive Performance

We investigated whether deterioration could be predicted using only values measured by wearable devices. The measurements of temperature, respiratory rate, pulse rate, and SpO_2_ were used, and the prediction performance of four classifiers based on an ensemble of decision trees was compared. Considering clinical utility, we aimed to predict deterioration 10 min and 8 h in advance. A comparison of the AUC values, accuracy, sensitivity, specificity, PPV, and NPV at optimized threshold values for each model is shown in Table 2. The average AUCs of the model predicting deterioration 10 min and the model predicting deterioration 8 h in advance were 0.992 and 0.815, respectively. Among the four classifiers, the XGB algorithm showed the best results, with the highest AUC values at both times.

### 3.3. Comparison of Different Feature Types and Model Development

**Figure 2:**
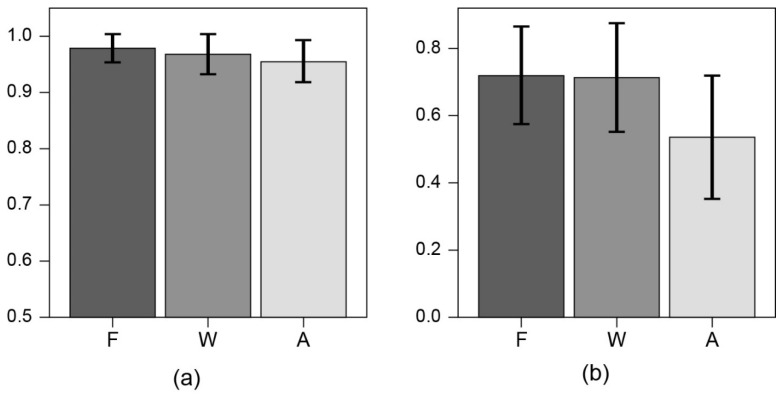
Comparison of the area under the receiver operating characteristic curve (AUC) of different feature combinations. Delong’s test was used for statistical performance comparison. Model prediction of fever (**a**) 10 min in advance, (**b**) 8 h in advance. F refers to the final model, W refers to the model using only features extracted from wearable devices, and A refers to the model using all features. The black vertical lines represent the standard deviation.

Overall, we found that among models based on ensembles of decision trees, the XGB model performed better than the other models for AUC. We also compared the performance of XGB models using various feature combinations to determine the performance difference between using only variables extracted from wearable devices and adding other self-reported symptom values. A total of 36 features were extracted, and we tested various combinations to find the optimal feature combination. Using only features obtained from the clinical questionnaire resulted in poor prediction performance. The comparison results between using all possible features, using only features extracted from wearable devices, and using some selected features are shown in Figure 2. Using fewer than 36 features gave better prediction performance. The model, including the selected variables, showed the best performance in the validation cohort. We selected nine features for the 10 min in advance model and eleven features for the 8 h in advance model and this model is the final model.

**Figure 3:**
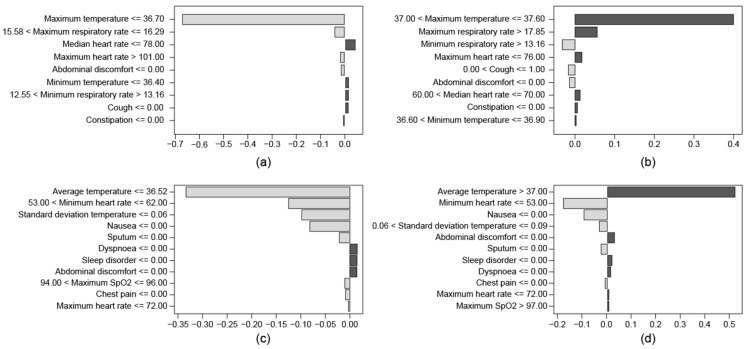
The importance of the final model using local interpretable model-agnostic explanation with optimal features. Negative values indicate parameters suggesting non-deterioration, and positive values indicate parameters suggesting deterioration. (**a**) is the 10 min deterioration prediction model with a non-deterioration case. (**b**) is the 10 min deterioration prediction model with a deterioration experienced case. (**c**) is the 8 h deterioration prediction model with a non-deterioration case. (**d**) is 8 h deterioration prediction model with a deterioration experienced case.

To assess the contribution of each optimal feature, the LIME method was applied to the final model, as shown in Figure 3. There is a difference in the features that provide information on the probability of deterioration depending on long and short prediction periods. For the 10 min deterioration prediction model, among nine features, the maximum temperature during the observation times was the most decisive (Figure 3a,b). Factors such as high respiratory rate, low heart rate, and coughing were of relatively low importance. On the other hand, in the 8 h deterioration prediction model, not only average temperature but also symptom information such as chest pain and nausea were decisive (Figure 3c,d). Additionally, we can see that although it is a respiratory disease, the virus is related to gastrointestinal symptoms. Moreover, the Pearson correlations were derived to provide insights into the relationship between individual features and model predictions, as shown in Table A3. The 10 min prediction model showed maximum temperature and maximum respiration rate as the main features, and the 8 h prediction model showed mean temperature and min heart rate. Similar results were yielded in the order shown in Figure 3. 

### 3.4. External Validation of the Proposed Models and Comparison of Their Predictive Performance

The characteristics of these collected data from the second clinical trial and the comparison with data from the first clinical trial are shown in Table A1 and Table A2 in Appendix A. Of the 181 patients, 122 experienced deterioration, and the average time with deterioration was 6.7 h, which was longer than that of the derivation cohort. Data were collected for a maximum of 6 days and a minimum of 3 days. The ages of the 181 patients ranged from 21 to 75 years, with a mean age of 37 years (SD 9.0). Patients wore the wearable devices for an average of 64.5 h (SD 33.2 h) during the isolation. In the questionnaire answered in the early stage of diagnosis, only fever showed a significant difference between the two groups. When comparing the characteristics of the derivation cohorts and external validation cohort, there was a significant difference in the presence or absence of cough, sputum, fever, sore throat, abdominal pain, and pain. The result of applying the previously trained model to the external validation cohort shows a similar pattern, but a slight performance decrease can be seen when predicting deterioration 8 h in advance in Table 3. The best combination explored previously also shows the highest AUC in both forecast times. It has the characteristic of showing a lower PPV compared with earlier. When calculating sensitivity for 122 patients who experienced a deterioration at least once in the external validation cohort, the highest sensitivity was 0.999, the lowest value was 0.530, the average was 0.888, and the standard deviation was 0.358. Additionally, we applied our final model to a variety of forecast time frames in addition to 10 min and 8 h are shown in Figure A2. The 10 min deterioration prediction model has a higher AUC than the 8 h model when the prediction time is longer than 7 h (Figure A2a). The 8 h deterioration prediction model has higher sensitivities than the 10 min model when the prediction time is longer than 3 h (Figure A2b).

## 4. Discussion

In this study, we proposed machine learning algorithms that predict the deterioration of patients with mild COVID-19 conditions 10 min and 8 h in advance with wearable device data. First, algorithms can be applied even if the device changes because we used the value of wearable devices, and it is versatile because it uses only characteristics that can be easily measured outside the hospital. Second, we found that the variables that need to be collected are different depending on the purpose of monitoring and the prediction interval to predict deterioration. Third, as we performed an external validation test in an environment where the algorithm would actually be used, we confirmed that both algorithms showed high accuracy. Fourth, utilizing the proposed algorithm will be useful for patient management and non-face-to-face treatment.

The strength of this study and the proposed algorithms is that anyone can participate, no matter what wearable device they have. It is useful because it targets the most common patients with mild symptoms and uses only the values from wearable devices. In fact, we used two different devices: a Seer Patch that attaches to the body and a Garmin device in the type of smartwatch. Since there is no need for difficult agreement with the manufacturer, such as adjusting sensor values, any device that can store and transmit values can be used to predict and monitor deterioration. Adding a specific algorithm to a medical device is difficult, but the devices used are not medical devices. Additionally, the number of users of wearable devices containing multiple sensors and functions is increasing every year. The deterioration monitoring applied in this study targeted the general public and used common devices, so it has a low barrier to entry for participation.

We found that features that should be considered vary depending on the time we want to predict deterioration in advance (Figure 3). This means that when applying the prediction model to an actual monitoring situation, the variables to measure and methods to measure will be different depending on the monitoring purpose. In particular, as the prediction time becomes longer, it is better to add symptom information that can be obtained from the patient’s answer, in addition to vital signs measured by wearable devices. This point can also be seen through the model’s additional validation results for a variety of forecast ranges. As we showed earlier, the longer the prediction time, the lower the prediction performance (Figure A2). We showed that the performance of the 8 h deterioration prediction model improves over the 10 min model as the prediction time increases, which supports the fact that longer prediction times require more clinical questionnaire information. In previous research studies, especially in cases where the time point was far from the event to be predicted, only symptom information was often collected by self-reports [21,38,39]. These results suggest that if the prediction interval is long, non-face-to-face treatment with medical staff will have the same effect as monitoring. However, this study showed that it is effective to use variables extracted from wearable devices even though we have long prediction times. This suggests that it can be an alternative to overcome difficulties that may arise during non-face-to-face treatment due to infectious diseases.

We evaluated predictive performance in the actual clinical setting. In a clinical setting, it is necessary to analyze data in a certain time period and derive corresponding results. Therefore, in most cases, the labels attached to the observation period are unbalanced. These collected data from the derivation cohort were preprocessed in a way that facilitated training the model, so it is difficult to say that the same results will be obtained when evaluated in a real clinical environment setting. To reflect these characteristics and effectively train the model, we preprocessed both sets of data from the two cohorts in different ways (Figure 1). This method is different from previous studies that made virtual data using oversampling methods. Furthermore, the model developed in this way showed consistent performance despite differences across study time points. The cohorts collected in this study were unparalleled, which led to significant differences in patient symptoms. For infectious diseases, the pattern of collected patients is likely to change due to virus mutations, and there is bound to be a difference between the time of model development and actual use, so it is important to maintain consistent performance. The variant of SARS-CoV-2 emerged in 2021, and the viruses that were prevalent in the two cohorts were different. Therefore, most patients in the derivation cohort were likely to have delta variants, and most patients in the external validation cohort were likely to have Omicron variants. There was a significantly different pattern in the number of patients presenting with symptoms including cough, sputum, fever, sore throat, and dyspnoea in the group with the Omicron variant (Table A2). Despite differences in the characteristics of the collected cohorts and evaluation process, the deterioration prediction models showed similar performance in both cohorts.

**Figure A1:**
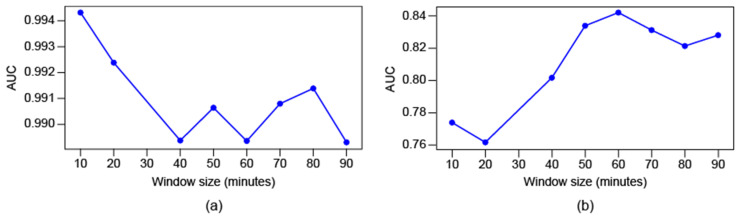
Prediction performance for various observation window lengths. The area under the receiver operating characteristic curve (AUC) values was the average value of multiple models. (**a**) The AUC when predicting deterioration in derivation cohort 10 min in advance. (**b**) The AUC when predicting deterioration in derivation cohort 8 h in advance.

**Figure A2:**
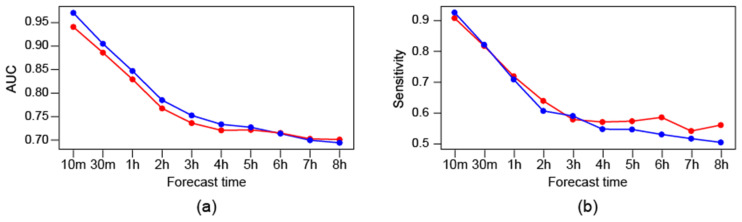
Performance of the final two models. The red line represents the result of using the 8 h in the advance model, and the blue line represents the result of using 10 min in the advance model. The *x*-axis represents the prediction time, m means minutes, and h means hours. (**a**) is the area under the receiver operating characteristic curve (AUC) of both final models. (**b**) is the sensitivity of both final models.

In addition, as many clinically mild cases occurred in COVID-19, people and material management are important to prevent the collapse of the medical system. Therefore, it is necessary to quickly identify patients at high risk of deterioration in the early stages of infection. We attempted to develop models with a short observation window to reduce the time to the first results. For the short prediction time model, prediction performance was not significantly affected by the window length, but for the long prediction time model, we found that using too short a window length has been shown to have poor prediction performance (Figure A1). The proposed model and analysis method can provide more objective and specific information to medical staff. If the medical staff received additional information from analyzing these data continuously measured by the patient at home, they would be able to make an accurate diagnosis through more detailed conversations during treatment, and the developed model helps speed up this process. It will be useful for both patients and medical staff when building a system to manage the general population during an infectious disease outbreak effectively.

This study had some limitations. First, our study only included people who were comfortable using electronic devices and communicating using them. Additionally, these results are based in Korea, and further evaluation and research using diverse data collected across other ethnicities and races is needed. Second, our fever prediction models were not able to provide information about how high fever will occur or how long it will last. Third, the fact that different patterns may appear depending on vaccination was not taken into consideration.

## 5. Conclusions

This study proposes an analysis method for the early prediction of deterioration that will occur after a certain period of time. We developed a model to predict deterioration after a short period of time and after a long period of time and evaluated it on additionally collected data. The algorithm with the best prediction performance was XGB, and we found that the factors considered important were different between predictions 10 min in advance and 8 h in advance. It will be useful to both patients and medical staff in establishing a system that can effectively manage the general public in the event of an infectious disease outbreak and provide better non-face-to-face treatment.

## Figures and Tables

**Table 1:** Demographic and health characteristics and comparison of deterioration and non-deterioration groups reported by quarantined coronavirus patients on the first day.

	Non-Deterioration (*n* = 22)	Deterioration (*n* = 28)	*p* Value
**Continuous variable, mean ± SD**			
Age	39.0 ± 15.141	40.107 ± 11.318	0.597
Systolic blood pressure	123.045 ± 14.147	124.214 ± 13.72	0.799
Diastolic blood pressure	82.545 ± 9.075	87.071 ± 9.718	0.068
Pulse rate	69.909 ± 11.309	77.679 ± 10.353	0.012
Respiratory rate	19.318 ± 7.779	17.643 ± 3.358	0.906
Temperature	35.973 ± 0.638	36.329 ± 0.546	0.041
Oxygen saturation	97.182 ± 1.259	97.071 ± 1.016	0.462
**Categorical variable, *n* (% total)**			
Cough	12 (54.55%)	16 (57.14%)	>0.999
Sputum	9 (40.91%)	17 (60.71%)	0.269
Fever	4 (18.18%)	4 (14.29%)	>0.999
Rhinorrhoea	8 (36.36%)	14 (50.0%)	0.498
Sore throat	11 (50.0%)	19 (67.86%)	0.323
Dyspnoea	1 (4.55%)	3 (10.71%)	0.785
Chest pain	1 (4.55%)	3 (10.71%)	0.785
Nausea	0 (0.0%)	2 (7.14%)	0.581
Vomiting	0 (0.0%)	0 (0.0%)	-
Abdominal discomfort	3 (13.64%)	3 (10.71%)	>0.999
Constipation	2 (9.09%)	4 (14.29%)	0.902
Diarrhea	2 (9.09%)	5 (17.86%)	0.634
Abdominal pain	2 (9.09%)	3 (10.71%)	>0.999
Pain	4 (18.18%)	13 (46.43%)	0.073
Sleep disorder	5 (22.73%)	8 (28.57%)	0.886

**Table 2:** Performance comparison of the four different tree-based models.

Forecast Range and Model	AUC	Accuracy	Sensitivity	Specificity	PPV	NPV
**10 min**						
RF	0.988	0.939	0.973	0.919	0.874	0.983
XGB	0.994	0.967	0.974	0.962	0.951	0.980
LGBM	0.992	0.961	0.951	0.967	0.944	0.972
CAT	0.992	0.959	0.951	0.963	0.938	0.972
**8 h**						
RF	0.814	0.820	0.674	0.911	0.826	0.817
XGB	0.842	0.804	0.700	0.887	0.834	0.786
LGBM	0.794	0.847	0.658	0.964	0.920	0.819
CAT	0.808	0.807	0.653	0.904	0.809	0.806

AUC, area under the receiver operating characteristic curve; PPV, positive predictive value; NPV, negative predictive value; RF, random forest; XGB, extreme gradient boosting; LGBM, light gradient boosting machine; CAT, CatBoost.

**Table 3:** Predictive performance in the external validation cohort using compact features.

Prediction Model	AUC	Accuracy	Sensitivity	Specificity	PPV	NPV
**10 min**						
W	0.970	0.917	0.931	0.916	0.430	0.995
C	0.572	0.733	0.399	0.756	0.100	0.949
A	0.968	0.929	0.912	0.931	0.498	0.993
F	0.973	0.921	0.926	0.920	0.468	0.994
**8 h**						
W	0.649	0.760	0.431	0.777	0.094	0.962
C	0.512	0.168	0.936	0.127	0.054	0.973
A	0.689	0.702	0.576	0.718	0.213	0.927
F	0.690	0.713	0.548	0.735	0.215	0.925

AUC, area under the receiver operating characteristic curve; PPV, positive predictive value; NPV, negative predictive value; W, model using only features extracted from wearable devices; C, model using only clinical questionnaire answers; A, model using all features; F, model using selected features.

## Data Availability

The data sets generated or analyzed during the current study are not publicly available in accordance with the hospital’s regulations, adhering to the National Privacy Act and relevant guidelines. However, if there is a reasonable request after review and approval of the institutional review board and the institutional data steering committee, it may be available from the corresponding author.

## Appendix A

**Table A1:** Demographic and health characteristics reported by quarantined coronavirus patients in the validation cohort on day one.

	Non-Deterioration (*n* = 59)	Deterioration (*n* = 122)	*p* Value
**Continuous variable, mean ± SD**			
Age	37.057 ± 9.601	35.949 ± 7.673	0.838
**Categorical variable, *n* (% total)**			
Cough	49 (83.05%)	102 (83.61%)	>0.999
Sputum	51 (86.44%)	100 (81.97%)	0.585
Fever	9 (15.25%)	51 (41.8%)	0.001
Rhinorrhoea	29 (49.15%)	73 (59.84%)	0.231
Sore Throat	42 (71.19%)	101 (82.79%)	0.109
Dyspnoea	0 (0.0%)	2 (1.64%)	0.818
Chest pain	6 (10.17%)	11 (9.02%)	>0.999
Nausea	2 (3.39%)	16 (13.11%)	0.074
Vomiting	0 (0.0%)	3 (2.46%)	0.553
Abdominal discomfort	6 (10.17%)	10 (8.2%)	0.874
Constipation	7 (11.86%)	11 (9.02%)	0.737
Diarrhea	6 (10.17%)	11 (9.02%)	>0.999
Abdominal pain	1 (1.69%)	3 (2.46%)	>0.999
Pain	28 (47.46%)	66 (54.1%)	0.497
Sleep disorder	8 (13.56%)	31 (25.41%)	0.104

**Table A2:** Comparison of demographic and health characteristics reported by quarantined coronavirus patients in the validation cohort and external validation cohort.

Characteristics	Year 1 (*n* = 50)	Year 2 (*n* = 181)	*p* Value
**Continuous variable, mean ± SD**			
Age	39.62 ± 13.005	36.696 ± 9.011	0.263
**Categorical variable, *n* (% total)**			
Cough	28 (56.0%)	151 (83.43%)	<0.001
Sputum	26 (52.0%)	151 (83.43%)	<0.001
Fever	8 (16.0%)	60 (33.15%)	0.029
Rhinorrhoea	22 (44.0%)	102 (56.35%)	0.164
Sore Throat	30 (60.0%)	143 (79.01%)	0.010
Dyspnoea	4 (8.0%)	2 (1.1%)	0.027
Chest pain	4 (8.0%)	17 (9.39%)	0.980
Nausea	2 (4.0%)	18 (9.94%)	0.299
Vomiting	0 (0.0%)	3 (1.66%)	0.833
Abdominal discomfort	6 (12.0%)	16 (8.84%)	0.688
Constipation	6 (12.0%)	18 (9.94%)	0.873
Diarrhea	7 (14.0%)	17 (9.39%)	0.494
Abdominal pain	5 (10.0%)	4 (2.21%)	0.035
Pain	17 (34.0%)	94 (51.93%)	0.037
Sleep disorder	13 (26.0%)	39 (21.55%)	0.634

**Table A3:** Pearson correlation coefficient between the features included in the final model and ground truth (patient deterioration).

Characteristics	Derivation	External Validation
**10 min**		
Maximum temperature	0.860	0.349
Maximum respiratory rate	0.400	0.099
Minimum respiratory rate	0.297	0.090
Maximum heart rate	0.299	0.031
Cough	0.126	0.025
Abdominal discomfort	−0.044	0.000
Heart rate median	0.438	0.059
Constipation	0.017	−0.002
Minimum temperature	0.161	0.237
**8 h**		
Average temperature	0.440	0.090
Minimum heart rate	0.421	0.069
Nausea	0.032	0.043
Standard deviation temperature	−0.011	0.012
Abdominal discomfort	−0.134	0.000
Sputum	0.113	0.014
Sleep disorder	−0.084	0.026
Dyspnoea	0.135	−0.023
Chest pain	0.145	−0.013
Maximum heart rate	0.262	0.057
Maximum SpO_2_	0.061	−0.015

To show the importance of explainable features, we obtain the Pearson correlation coefficient between features included in the final model and ground truth.
